# Reply to Pernet et al.: Mesocosm experiment reveals the carbon removal mechanisms in oyster farming

**DOI:** 10.1073/pnas.2536882123

**Published:** 2026-02-27

**Authors:** Shuang-Lin Dong, Shuh-Ji Kao, Xiang-Li Tian, Xue-Wei-Jie Chen, Zhou Zhang, Miao-Jun Pan, Yun-Wei Dong

**Affiliations:** ^a^Key Laboratory of Mariculture of Ministry of Education, Fisheries College, Ocean University of China, Qingdao 266003, China; ^b^State Key Laboratory of Marine Resource Utilization in South China Sea, Hainan University, Haikou 570228, China; ^c^Qingdao Marine Science and Technology Museum, Qingdao 266100, China

We thank Pernet et al. for their commentary (1) on our study (2). Below, we clarify key points within a concise framework.

1. Mesocosm design isolates biological signals
  Field carbon budgets are confounded by physical advection and mixing. Our mesocosms provided controlled, mass-balanced accounting of carbon fluxes, isolating oyster-mediated effects from hydrodynamic noise. The comparative control—no imposition of an experimental variable (3)—ensures that observed enhancements in carbon influx, sedimentation, and autotrophy are directly attributable to oysters.
2. Stocking density reflects ecosystem-scale reality
  In open coastal farms, water renewal means the effective water volume supporting oysters is larger than the farm footprint. Literature-calculated effective densities of oyster farms range from 0.09 to 12.50 g m^−3^ (4–7). Our experimental density (~33 g m^−3^ in static volume) aligns with this range when considered at ecosystem scale, confirming that realistic densities can drive net carbon removal.
3. Sediment carbon deposition is the essential first step toward sequestration
  We agree that long-term burial requires multidecadal stability. However, sequestration begins with enhanced carbon transfer to sediments. Our study quantified a significantly greater depositional Sedimentary Organic Carbon in oyster systems—a measurable signature of the “carbon-concentrating pump.” Empirical evidence shows that such deposition can outpace remineralization, leading to net accumulation over years (8, 9).
4. Shell disposal is a management issue, not a fixed carbon cost
  Our study focused on in situ ecosystem dynamics. Postharvest shell fate—incineration vs. reuse in restoration, agriculture, or construction—is a policy and circular-economy choice, not an inherent flaw. Life-cycle assessments indicate that some bivalve systems can be net carbon sinks (10, 11).
5. Field patterns confirm ecosystem-scale carbon processing and uptake
  Inside farms, chlorophyll is reduced by grazing, and dissolved oxygen is often undersaturated due to elevated respiration fueled by imported and internally produced organic matter. This reflects the system’s role as an intensified carbon reprocessing zone. At the farm fringe, elevated chlorophyll results from nutrient export, stimulating peripheral primary production. This spatial uncoupling—grazing and respiration inside, enhanced primary production outside—is a hallmark of ecosystem engineering and confirms that oyster farming enhances ecosystem-scale carbon turnover and drawdown, as evidenced by our whole-system mesocosm budgets.
6. Oyster farming acts as a carbon-concentrating and recycling pump
  Oysters concentrate and reprocess carbon, redirecting it to biomass, rapidly sinking biodeposits (SOC), and recycled nutrients that stimulate further primary production. This positive feedback enhances CO_2_ drawdown capacity, whether carbon originates inside the system or is imported.

## Conclusion

Our mechanistic evidence shows oyster farming enhances coastal carbon removal. Further research on long-term burial and full life-cycle assessment is needed, but dismissing this nature-based strategy overlooks its potential to integrate climate mitigation with food security and restoration.
